# Impaired liver regeneration is associated with reduced cyclin B1 in natural killer T cell-deficient mice

**DOI:** 10.1186/s12876-017-0600-2

**Published:** 2017-03-23

**Authors:** Ami Ben Ya’acov, Hadar Meir, Lydia Zolotaryova, Yaron Ilan, Eyal Shteyer

**Affiliations:** 1 Liver Unit, Hebrew University-Hadassah Medical Center, Jerusalem, Israel; 2 Department of Pediatrics, Hebrew University-Hadassah Medical Center, Jerusalem, Israel

**Keywords:** Liver regeneration, NKT cells, CD1d, CyclinB1, Interleukin-6

## Abstract

**Background:**

It has been shown that the proportion of natural killer T cells is markedly elevated during liver regeneration and their activation under different conditions can modulate this process. As natural killer T cells and liver injury are central in liver regeneration, elucidating their role is important.

**Methods:**

The aim of the current study is to explore the role of natural killer T cells in impaired liver regeneration. Concanvalin A was injected 4 days before partial hepatectomy to natural killer T cells- deficient mice or to anti CD1d1-treated mice. Ki-67 and proliferating cell nuclear antigen were used to measure hepatocytes proliferation. Expression of hepatic cyclin B1 and proliferating cell nuclear antigen were evaluated by Western Blot and liver injury was assessed by ALT and histology.

**Results:**

Natural killer T cells- deficient or mice injected with anti CD1d antibodies exhibited reduced liver regeneration. These mice were considerably resistant to ConA-induced liver injury. In the absence of NKT cells hepatic proliferating cell nuclear antigen and cyclin B1 decreased in mice injected with Concanvalin A before partial hepatectomy. This was accompanied with reduced serum interleukin-6 levels.

**Conclusions:**

Natural killer T cells play an important role in liver regeneration, which is associated with cyclin B1 and interleukin-6.

## Background

The liver maintains a steady mass balanced by cell gain and cell loss. The tight and regulated mechanisms guaranteeing this steady state are well shown in liver regeneration. The liver is comparatively rich in NKT cells implying of their important role in liver biology [1]. NKT cells are a heterogeneous group of T cells that recognize various lipid antigens presented by the non-classical MHC class I-like molecule CD1 [2, 3].

Accumulating data suggests that the role of NKT cells in the pathogenesis of liver disease is complex and that these cells likely to play different roles especially given the existence of multiple types of NKT cells, their associated cytokines and the divergence of their foreign and self-antigen lipids [4, 5]. Several lines of evidence indicate that NKT cells play an important role in triggering and promoting liver regeneration. After partial hepatectomy (PH) the number of NKT was shown to increase and NKT cells were able to mediate cytotoxicity against regenerating hepatocytes [6, 7].

Furthermore, activation of NKT cells by the powerful ligand α-galactosylceramide has been shown to accelerate liver regeneration after PH through the TNF and FasL-mediated pathway, and NKT cell-deficient mice (CD1d-/-) mice that were not treated with α-galactosylceramide showed decreased hepatocyte mitosis after PH [8]. Likewise, liver regeneration was impaired in mice pre-treated with anti NK1.1 antibodies [9].

Dong and colleagues demonstrated reduced liver regeneration in HBV-tg mice that was associated with activated NKT cells and their IFN-γ production. They further showed that blockade of CD1d receptor by monoclonal anti CD1d antibody, restored the impaired liver regeneration in these mice [10]. Concanavalin A (Con A), a plant lectin, is widely used to induce in mice rapid, severe and dose-dependent hepatitis and subsequent liver injury [11, 12]. NKT cells mediate the liver injury in the Con A immune mediated hepatitis model. CD1d-/- mice that lack NKT cells or mice with reduced hepatic NKT cell numbers, exhibit less severe liver pathology than wild-type controls in response to ConA [13, 14]. The adoptive transfer of hepatic NKT cells to NKT deficient mice restores ConA-mediated hepatitis [14].

The mechanisms by which NKT cells contribute to hepatic inflammation and liver damage in Con A hepatitis have been intensively studied and the regenerative processes were shown to be essential to restore liver function [15], but mechanisms that affect impaired liver regeneration were not always addressed. Wei-Hua et al showed that impaired liver regeneration in mice with hyperhomocysteinemia (HHcy) was also correlated with reduced cyclins and elevated expression of p53 and p21^Cip1^ [16]. In contrast, p21^Cip1^ knockout mice display accelerated hepatocyte proliferation after PH through G_1_ phase. In these mice up regulation of Cyclin A, proliferating cell nuclear antigen (PCNA) and Cyclin D1 were observed [17]. In NKT deficient mice impaired liver regeneration was not investigated regarding cell cycle modifiers. NKT cells are thus involved both in mediating the liver injury and in the liver repair process and regeneration. As NKT cells are required to initiate liver injury, the determination of their direct role in this response can be answered only by using selective depletion of NKT cells following the initiation of liver injury that is required to fully elucidate their role in liver regeneration. Another important feature of liver regeneration is the increased production of IL-6 as in other acute liver damages [18] and some studies have also shown that this cytokine plays an important role in protection against liver injury during liver regeneration [19, 20].

In the current study we aimed to investigate the effect of pretreatment with a non-lethal dose of ConA on subsequent liver regeneration, regarding NKT cells, thus providing new insight into the roles of Cyclin B1, p21 and IL-6.

## Methods

### Animals

C57Bl/6 male mice (9–10 weeks of age) were purchased from Harlan Laboratories (Jerusalem, Israel). C57BL/6- CD1d–/– mice were kindly provided by Luc Van Kaer (Vanderblit University, Nashville, TN, USA). Mice were bred and maintained in specific pathogen-free conditions. For experiments male mice (9–10 weeks of age) were used. All animals were provided *ad labium* access to a commercial rodent diet and water. The joint ethics committee (IACUC) of the Hebrew University and Hadassah Medical Center approved the study protocol for animal welfare. The Hebrew University is an AAALAC International accredited institute.

### ConA treatment and CD1d targeting

Wild type or CD1d-/- mice were injected intravenously with either vehicle (50 mmol/L Tris, 150 mmol/L sodium chloride and 4 mmol/L calcium chloride) or with 10 mg/kg ConA (MP Biomedicals, Ohio, USA) 4 days before PH. Depletion of NKT cells was carried out by administration of anti-CD1d mAb, which can effectively block CD1d receptor [21]. Control mice were injected isotype control antibody (rat IgG2b). Antibodies (eBioscience) were administered i.v. at a dose of 50 μg/mouse 1 h before ConA injection. Three-four animals were included in each group.

### Partial hepatectomy

All mice, treated or untreated with ConA, were subjected to 70% PH under isoflurane anesthesia as described [22] by removal of the median and left lateral lobes. Three hours or 48 h after PH mice were sacrificed and then serum and tissues were collected.

### Evaluation of liver regeneration

We used Ki-67 to evaluate the proliferation of hepatocytes 48 h after PH. After sacrifice, formalin-fixed, paraffin-embedded liver samples were cut in 5-μm-thick sections for staining. For immunostaining, sections were deparaffinized, treated in a pressure cooker with citrate buffer and incubated with rabbit anti-mouse Ki-67 (Abcam, Cambridge, UK) diluted 1:100 overnight at 4 °C. After washing the sections were incubated with Mach-3 mouse HRP-polymer (BioCare Medical, Pike Lane, CA, USA), then rinsed in diaminobenzidine (DAB) containing 0.02% hydrogen peroxide. Finally sections were counterstained with hematoxylin and examined under a light microscope. The number of Ki-67 positive hepatocytes was determined in liver tissues by counting 8 fields at X100 magnification.

### Serum ALT measurements

Blood was collected from all animals by cardiac puncture 48 h after PH. Levels of serum alanine aminotransferase (ALT) activity were measured by the Reflovet Plus system (Roche Diagnostics, Mannheim, Germany).

### SDS PAGE and Western blot analysis

Whole liver protein extracts were prepared by homogenizing frozen tissue in a buffer containing 50 mM Tris-HCl (pH 8.0), 150 mM NaCl, 1 mM EDTA, 1% Triton X-100, protease and phosphatase inhibitors (Sigma, Rehovot, Israel), followed by centrifugation at 4000 × *g* for 10 min at 4 °C. Protein concentration was determined by Bradford assay using the Bio-Rad protein assay kit (Bio-Rad Laboratories, Rehovot Israel). In general, 50–60 μg of total liver protein was separated on 10% sodium dodecyl sulfate-polyacrylamide gel electrophoresis (SDS-PAGE) and transferred to polyvinylidene difluoride membranes (Millipore, Bedford, MA). All membranes were stained with Ponceau S to confirm transfer of protein. After blocking with 5% nonfat dry milk in Tris-buffered saline- Tween 20, membranes were incubated overnight at 4 °C with rabbit polyclonal PCNA or p21 or cyclin B1 (Cell Signaling Technology, Beverly, MA, USA). β-actin (Abcam, Cambridge, UK) was used as loading control. Membranes were then incubated with secondary horseradish peroxidase-conjugated anti-rabbit IgG antibody (Dako A/S, Glosrtup, Denmark). Subsequently, specific bands were visualized using the EZ-ECL chemiluminescence detection kit (Biological Industries, Israel). Images were captured using a lumino-image analyzer (LAS-3000; Fujifilm, Tokyo, Japan) and densitometry was performed using EZQuant-Gel (EZQunat Ltd, Tel Aviv, Israel).

### Determination of serum IL-6 levels

Blood samples were obtained by cardiac puncture of all animals 48 or 3 h after PH. After 30 min of coagulation at room temperature, serum was separated and stored at −80 °C until assay. Serum levels of IL-6 were determined in various time points by sandwich ELISA using a commercial kit according to the manufacturer’s instructions (Quantikine, R&D Systems, Minneapolis, MN, USA).

### Statistics

Results are expressed as the mean ± SE. Results were assessed using student *t* test. *P* < 0.05 was considered statistically significant.

## Results

### Liver regeneration after PH is inhibited in the absence of type I NKT cells

**Fig. 1:**
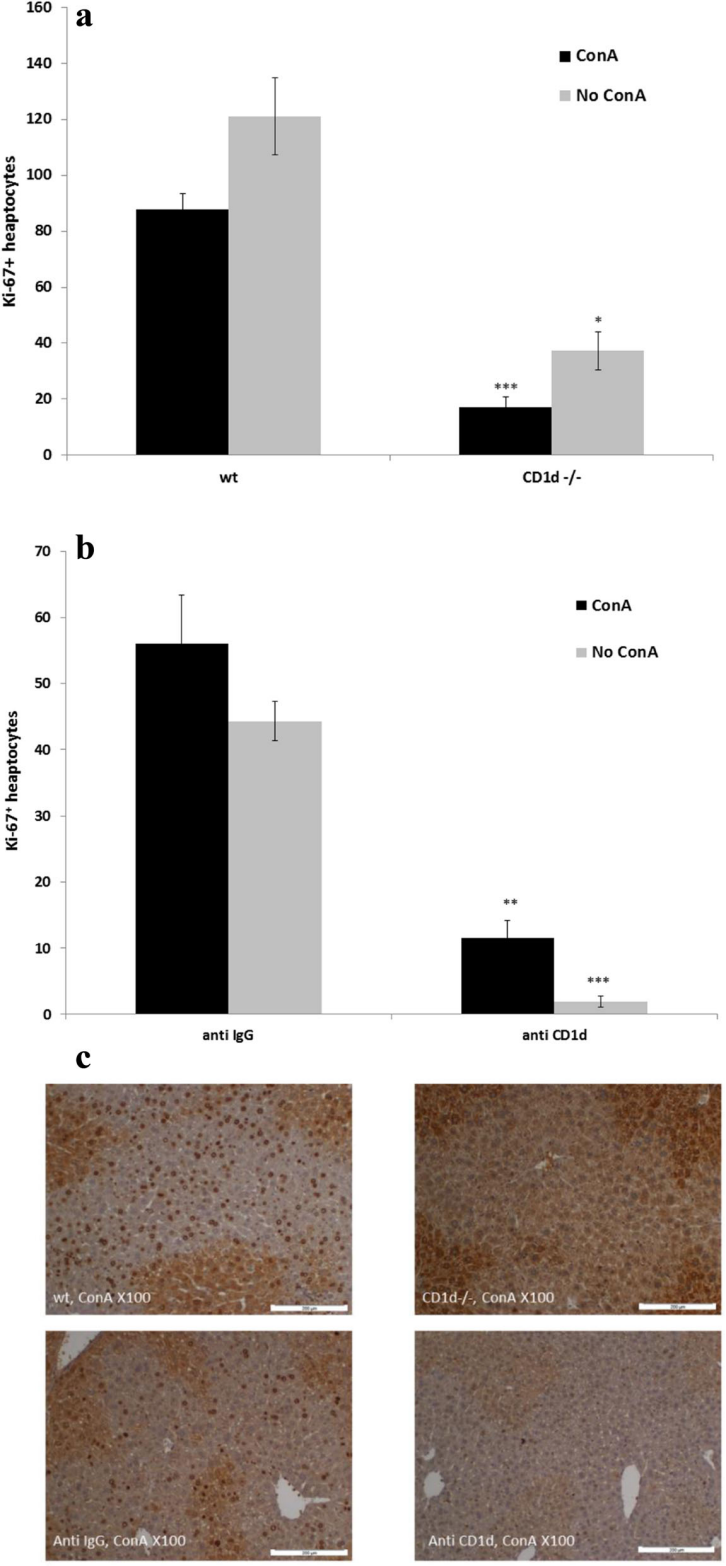
The effect of ConA on liver regeneration in the absence of NKT cells. **a** ConA was intravenously injected to CD1d-/- or to wild type mice 4 days prior to 70% PH. In other the experiments vehicle was injected instead of ConA in the same setting. Hepatocellular proliferation was measured by Ki-67 48 h after PH. **b** Anti CD1d or isotype control antibodies were intravenously injected 1 h before ConA administration 4 days prior to 70% PH. In the other experiments vehicle was injected instead of ConA in the same setting. Data represent means ± standard error of a representative experiment. *N* = 3 mice or more per condition. All experiments were repeated 3-4 times. In CD1d-/- experiments, * *p* < 0.005; *** *p* < 0.00005, relative to wt. In anti CD1d experiments, ** *p* < 0.0005; *** *p* < 0.00005, relative to isotype control. **c** Immunochemistry of Ki-67 positive hepatocytes, 48 h after PH from ConA-treated liver of wt and CD1d-/- mice (*upper panel*) and from isotype control and anti CD1d-treated mice (*lower panel*). Representative photomicrographs at X100 magnification are presented. Scale bars: 200 μm

To better define the role of NKT cells in liver regeneration, we used ConA to impair this process, examining the effects of ConA-mediated NKT cell activation on liver regeneration. The ConA model represents a well-established immune mediated liver disease, which is NKT cell mediated [14]. As hepatocyte damage can lead to compensatory liver regeneration, we aimed to examine how pre-treatment with ConA can affect liver regeneration in regard to NKT cells presence and activity. To address that question two methods were employed. The first was to use CD1d-/- mice that are deficient in NKT cells. Figures 1a and c presents the results of liver regeneration measured by Ki-67 in livers of wild type (wt) and in CD1d -/- mice. It can be seen that regardless of ConA administration liver regeneration was dependent on the presence of NKT cells. Four days after ConA administration liver regeneration after PH in CD1d -/- mice was significantly decreased compare to wt mice. The same results were obtained in vehicle (no ConA) experiments. The second method we used was by targeting the CD1d receptor using anti CD1d antibodies. This method to block NKT activity was used by several studies [21, 23]. Fig. 1b demonstrates that liver regeneration was significantly reduced in anti CD1d-treated mice, compared to isotype control-treated mice. Hepatocytes injury caused by pre-treatment of ConA did not affect these results. However, in these experiments the level of regeneration was higher in ConA experiments compared to non-ConA experiments. Similar results to Ki-67 staining were obtained with bromodeoxy uridine (BrdU) incorporation and mitotic bodies counts (data not shown).

Figure 1c, upper panel, shows Ki-67 staining of livers taken from wt and CD1d-/- mice 48 h after PH that were pre-treated with ConA. Similar histological results were observed in mice that were injected with anti CD1d antibodies and were compared to of isotype control IgG-treated mice (lower panel). Taking together these results demonstrate the importance of type I NKT cells in liver regeneration.

### The effect of impaired liver regeneration on cell cycle regulators

**Fig. 2:**
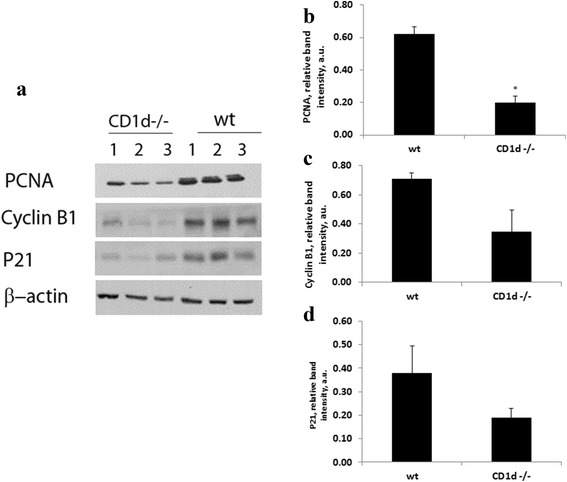
Expression patterns of hepatic PCNA, P21 and Cyclin B1 during liver regeneration from ConA-challenged wild type and CD1-/- mice. **a** Western blotting analysis of hepatic remnant lysates, as described in materials and methods, was performed 48 h after PH using anti PCNA, anti p21 and anti cyclinB1. β-actin was used as loading control. The illustrated bands are representative of 3 mice per group. **b** Densitometry of PCNA **c** Densitometry of Cyclin B1 **d** Densitometry of p21. Bars are means ± standard error values of intensity for individual bands that were quantified using EZQuant-Gel densitometry software, and expressed relative to β-actin, as a measure of protein relative abundance in the different liver samples. * *p* < 0.02, relative to wt. a.u.: arbitrary units

**Fig. 3:**
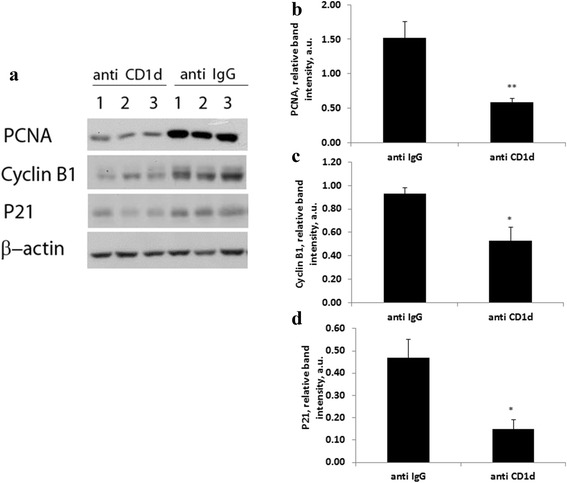
Expression patterns of hepatic PCNA, P21 and Cyclin B1 during liver regeneration from ConA-challenged isotype control and anti CD1d-treated mice. **a** Western blotting analysis of hepatic remnant lysates, as described in materials and methods, was performed 48 h after PH using anti PCNA, anti p21 and anti cyclinB1. β-actin was used as loading control. The illustrated bands are representative of 3 mice per group. **b** Densitometry of PCNA **c** Densitometry of Cyclin B1 **d** Densitometry of p21. Bars are means ± standard error values of intensity for individual bands that were quantified using EZQuant-Gel densitometry software, and expressed relative to β-actin, as a measure of protein relative abundance in the different liver samples. * *p* < 0.05, ** *p* < 0.02 relative to anti IgG. a.u.: arbitrary units

Expression of several cyclins are correlated with hepatocyte proliferation after PH [17, 24, 25]. In our study we were interested to find whether reduced regeneration was associated with decreased expression of cell cycle modifiers. So we further analyzed how impaired hepatocyte proliferation affected up-stream cell signaling effectors. Wild type and CD1d-/- mice were injected with ConA and 4 days later 70% PH was carried out and mice were sacrificed 48 h later. Total proteins were isolated from livers and subjected to immunoblotting with antibodies against PCNA, cyclin B1 and p21 or β-actin (Fig. 2a). An analysis of liver cell proliferation by PCNA expression in hepatocytes previously exposed to ConA, revealed 3 -fold less proliferation 48 h following PH in CD1d-/- mice compared with wt mice (Fig. 2a, b). The hepatic expression of cyclin B1 and p21 was also considerably lower in CD1d-/- mice; however differences did not reach statistical significance (Fig. 2c and d). Figure 3a demonstrate similar results that were observed in livers from anti IgG and anti CD1d-treated mice. Hepatic expression of PCNA, cyclin B1 and p21 48 h following PH in anti CD1d-treated mice that were previously exposed to ConA; was considerably lower than in control-anti IgG treated mice (Fig. 3a). Injection of anti CD1d antibodies caused significant decrease in the expression of these cell cycle regulators (Fig. 3b, c and d).

### ALT level after impaired PH is decreased in the absence of type I NKT cells

**Fig. 4:**
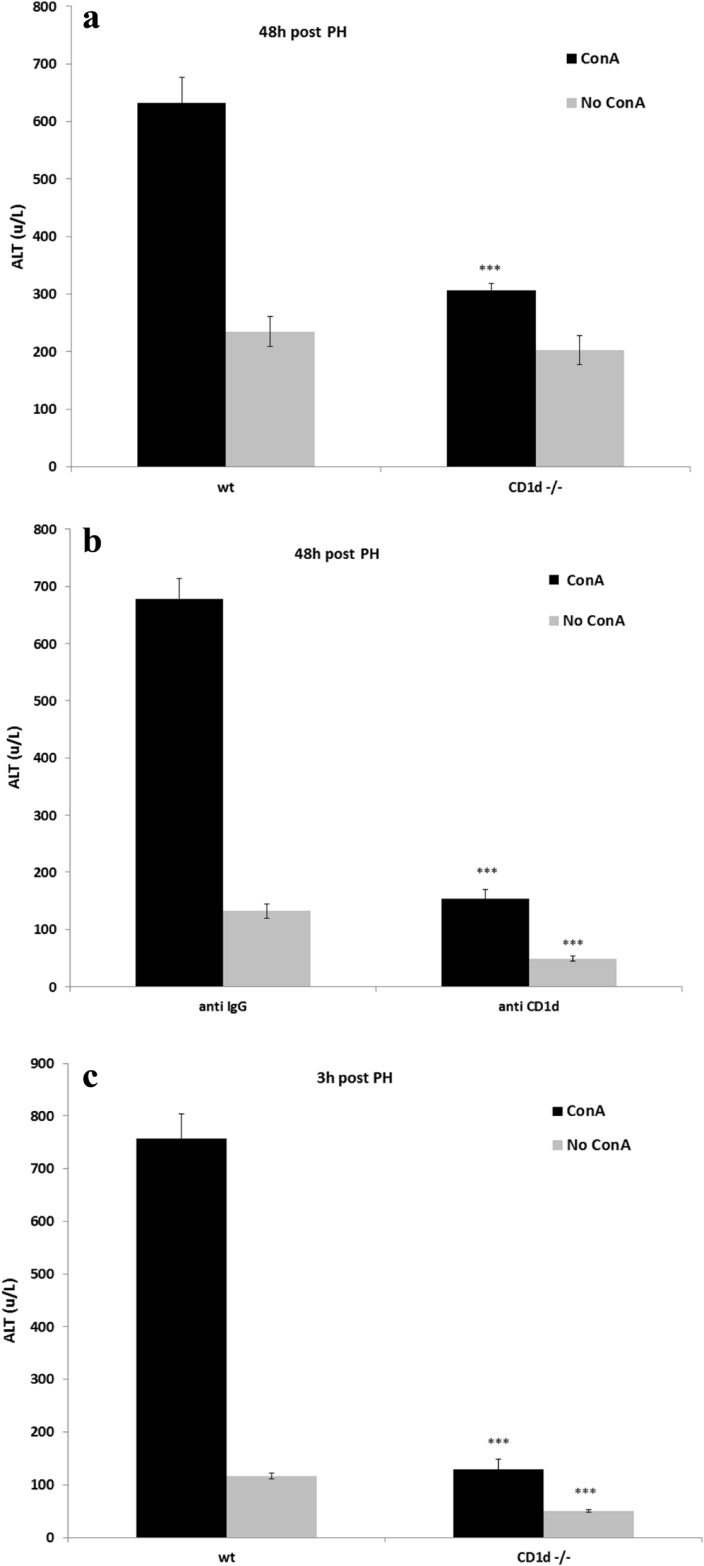
The effect of ConA on liver injury after partial hepatectomy. **a** ConA was intravenously injected to CD1d-/- or to wild type mice 4 days prior to 70% PH. In other experiments vehicle was injected instead of ConA in the same setting. Serum ALT levels were measured 48 h after PH. **b** Anti CD1d or isotype control antibodies were intravenously injected 1 h before ConA administration 4 days prior to 70% PH. In other experiments vehicle was injected instead of ConA in the same setting. Serum ALT levels were measured 48 h after PH. **c** As in A, but ALT levels were measured 3 h after PH. Bars are means ± standard error values. *N* = 9 mice per condition. ***, *p* < 0.00005, relative to wild type or IgG-isotype control

Before hepatocytes undergo necrosis after ConA treatment, ALT is released from these cells into blood stream, resulting in abnormally high serum levels of this enzyme. In our study we were interested to examine liver damage after PH in mice that were challenged by ConA 4 days prior to PH. Figure 4a shows the results of ALT levels that were measured 48 after PH in mice that were previously administered ConA. Average ALT levels 48 h after PH reached 633u/L in wt mice. In contrast, ALT levels in CD1d-/- mice were significantly lower by 50%. In wt and CD1d-/- mice that were not exposed to ConA prior to PH, ALT levels were comparable. Similar results were observed 48 h following PH in anti IgG and anti CD1d-treated mice that were previously exposed to ConA (Fig. 4b). Average ALT levels reached 678u/L in wt mice, in contrast to average of 154u/L of ALT measured in mice injected with anti CD1d antibodies. When mice were not subjected to ConA ALT levels remained low in mice that were treated with isotype control IgG antibodies and even significantly lower in anti CD1d-treated mice. Figure 4c shows that ALT levels measured 3 h after PH in wt mice previously exposed to ConA, were significantly elevated compare to CD1d-/- mice. However, when mice were not treated with ConA prior to PH, the ALT levels measured in CD1d-/- mice was even lower than in wt mice. Interestingly, the level of ALT enzyme in wt mice previously exposed to ConA, that were measured 3 h after PH were similar to those measured 48 h after PH. Taking together these results demonstrate that ALT rise after PH in mice treated with ConA is caused by ConA insult and depend in type I NKT cells.

### NKT cells affect the secretion of IL-6 in liver regeneration

**Fig. 5:**
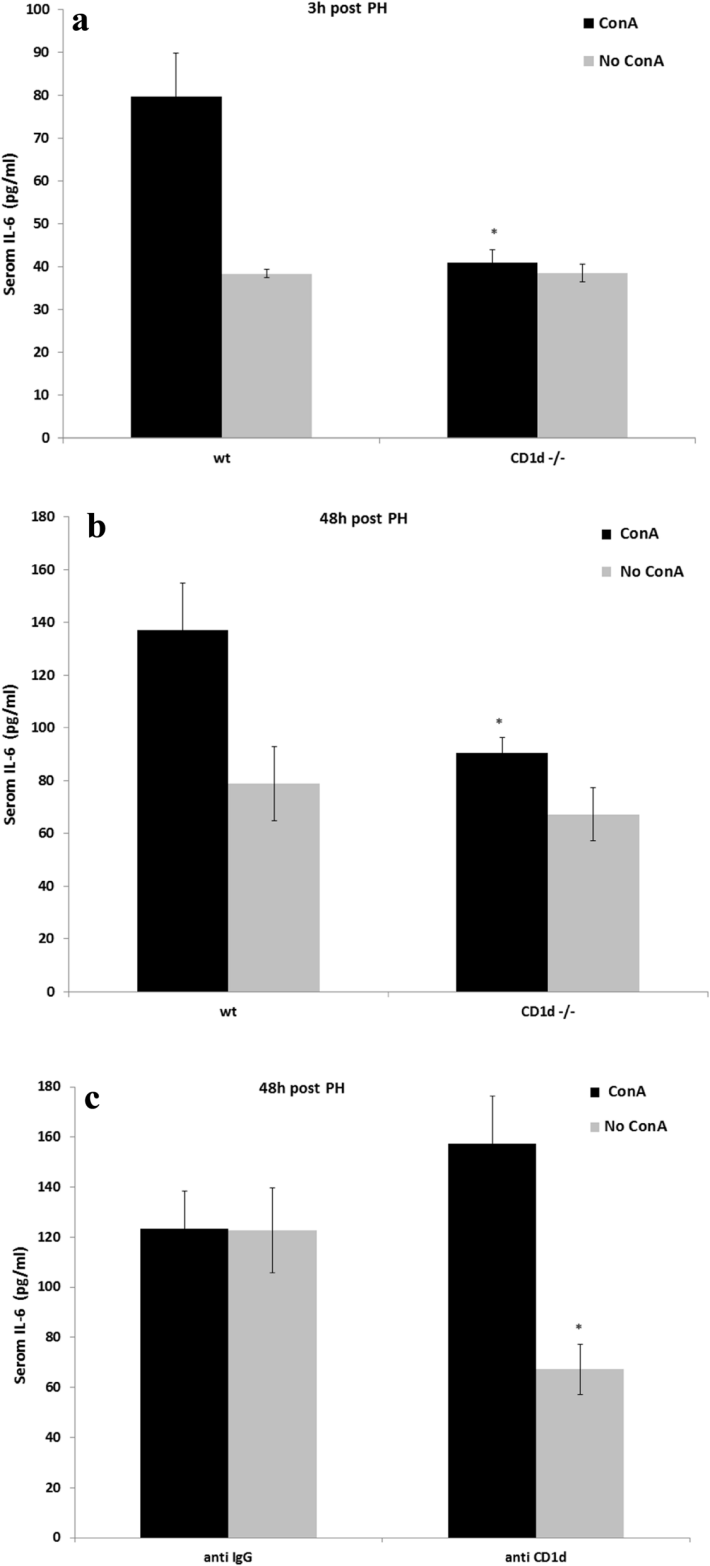
The effect of partial hepatectomy on serum IL-6 secretion after ConA challenge. **a** ConA was intravenously injected to CD1d-/- or to wild type mice 4 days prior to 70% PH. In the other experiments vehicle was injected instead of ConA in the same setting. Serum IL-6 levels were measured 3 h after PH by ELISA (**a**). **b** As in A, but IL-6 levels were measured 48 h after PH. **c** Anti CD1d or isotype control antibodies were intravenously injected 1 h before ConA administration 4 days prior to 70% PH. In the other experiments vehicle was injected instead of ConA in the same setting. Serum IL-6 levels were measured 48 h after PH. Bars are means ± standard error values. *N* = 9 mice per condition. * *p* < 0.05 or * *p* < 0.01, relative to wild type or IgG-isotype control, respectively. ***, *p* < 0.001, relative to wild type or IgG-isotype control

IL-6 is a key cytokine of liver regeneration that is also elevated during liver disease [26]. Therefore we wanted to determine how the levels of this cytokine were affected in ConA-impaired liver regeneration, in regard to NKT cells. Figure 5a shows the results of serum IL-6 levels 3 h after PH, from wt and in CD1d-/- mice that were exposed to ConA. Serum IL-6 levels in CD1d-/- mice were significantly lower in CD1d-/- mice compare to wt mice, 3 h after PH. However, when mice were not exposed to ConA, similar levels of serum IL-6 levels were recorded in wt and in CD1d-/- mice, indicating that IL-6 levels were affected only in wt mice. Similar results, with or without ConA, were obtained when serum IL-6 levels were measured 48 h after PH (Fig. 5b). However, serum IL-6 levels 48 h after PH in mice previously exposed to ConA and treated with anti CD1d antibodies was higher than in controls. But when mice were not exposed to ConA prior to PH the treatment with anti CD1d antibodies lowered serum IL-6 levels by 45% (Fig. 5c). Taken together, after PH CD1d-/- mice previously exposed to ConA, exhibited significantly lower IL-6 levels compare to wt mice, these results correlate with reduced liver regeneration in these mice.

## Discussion

Although NKT cells have been shown to be involved in immune mediated diseases [27], asthma [28], antitumor immune responses [29] and inflammatory liver disease [14, 30, 31], their role in liver regeneration is not fully elucidated. In the current study we aimed to explore one aspect of NKT in liver regeneration.

To investigate the contribution of NKT cells to liver regeneration we first choose to activate NKT cells by ConA. ConA is a plant lectin that induces hepatitis in a well-characterized model of NKT cell-mediated liver disease [32]. Administration of moderate doses of ConA is a well-accepted tool to activate NKT cells. In 8–15 h significant rise in aminotransferases is observed but at later time points, usually after 48 h, liver function is reconstituted [33]. Previous studies have demonstrated the critical contribution of NKT cells to ConA-induced hepatitis by showing that CD1d deficient mice were highly resistant to ConA injection compared to wt mice [13, 14]. In the current study we showed in CD1d-/- mice and in anti CD1d-treated mice by well-accepted mitotic markers (Ki-67 and PCNA) that hepatocyte proliferation 48 h after PH is markedly reduced. In contrast, CD1-/- mice on balb.c background have been shown to demonstrate almost normal liver regeneration after PH [9, 34]. It is important to stress out that NKT cell numbers are highly variable between mouse strains [35].

Early enhanced regeneration due to ConA pretreatment was attributed to the disappearance of NKT by activation-induced cell death (AICD). Within the current model, ConA induces the rapid activation and depletion of NKT cells, which serve to initiate the injury response. Forty eight hours or more after PH NKT cells can repopulate the liver and are therefore capable of contributing to the regenerative response [36]. Huang et al showed that ConA pretreatment did not accelerate liver regeneration 48 h after PH, although at earlier time points ConA significantly induced liver regeneration rate. In this study NKT cells disappeared 24 h after ConA stimulation which was the time point of PH [15]. We observed that pre-treatment with ConA 4 days before PH revealed lower hepatic proliferation 48 h after PH, compared to non-ConA experiments. Similar observations were obtained by Hines and colleagues that showed under the same experiment setting 40% reduced cell proliferative response after ConA challenge. Four days after ConA administration NKT cells repopulate the hepatic parenchyma [36]. Collectively the negatively regulated role of activated NKT cells diminished along the restoration of NKT cells 48 h after PH. Tekada et al first demonstrated the critical contribution of NKT cells to ConA-induced hepatitis by showing that CD1d deficient mice were highly resistant to ConA injection compared to wt mice [14]. Nevertheless, the role of NKT cells in liver regeneration is less clear. Impaired liver regeneration by using ConA may provide a tool of exploring their role. We showed significant decreased liver injury in CD1d-/- mice and in anti CD1d-treated mice that were pre-treated with ConA 4 days before PH. High ALT levels 3 h and 48 h after PH are completely attributed to ConA treatment, as without ConA ALT levels in the 2 models were similar to wt or isotype-control mice. When ConA was not applied, targeting CD1d receptor by mAb was reflected by significantly reduced hepatic proliferation, which may be associated with possible blunting of CD1d receptors on hepatocytes. One cannot rule out that impaired liver regeneration is associated with reduced liver injury caused by the absence of NKT cells. The expression of several cyclins is correlated with hepatocyte cell cycle progression after PH [17, 24, 25]. Hines and colleagues, who attributed the mechanisms of ConA pre-treatment to the modulation of oval cells, have also studied the effect of impaired liver regeneration on cell cycle. They showed that in ConA pretreated mice the expression of Cyclin D1 was reduced 6 and 24 h after PH and of Cyclin E after 48 h. They further showed that phosphorylated Stat3 and IL-6 were reduced in ConA-induced hepatitis, whereas p21 and Smad2 increased. These findings are in agreement with a recent report showing that depletion of NK and NKT cells by anti NK.1.1 antibody resulted in reduced BrdU uptake along with decreased expression of PCNA and Cyclin D, 48 h after PH [9]. However, under the context of NKT deficiency cell cycle modifiers were not thoroughly investigated. In our study we show decreased expression of hepatic Cyclin B in CD1d-/- mice and in anti- CD1d-treated mice 48 h after PH. These results were observed in impaired liver regeneration with ConA or without ConA (data not shown). Surprisingly, we found that the levels of p21 in CD1d1-/- mice and in anti- CD1d treated mice were lower compared to wt and isotype control mice. Although this level expression was measured 48 h after PH and not earlier, we cannot rule other mechanistic options. Indeed, p21 was demonstrated as a key inhibitor of G_1_ to S phase progression in hepatocytes [37] and its expression inhibits several genes involved in cell cycle [38], nevertheless, recent discoveries suggest that p21 has additional activities that are unrelated to its function as CDK inhibitors, especially in light of the identification of new targets as well as evidence of Cip/Kip cytoplasmic re-localization [39–41]. However, more investigation is needed to explore the connection between NKT deficiency and p21.

The effect of IL-6 on various forms of liver injury, including ConA and liver regeneration has been well documented. In our study we found that the reduced hepatocyte proliferation in the absence of NKT cells was accompanied with significant decreased levels of serum IL-6 levels 3 and 48 h after PH. This observation was regardless of ConA challenge. Accordingly, in mice treated with NK1.1 antibody, IL-6 mRNA levels following PH were blunted significantly [9]. Also in NKT cell-deficient mice, the diminished ConA-induced liver injury was restored by the adoptive transfer of liver mononuclear cells or NKT cells from wild-type mice, but not from IL-6-treated mice [42]. In the study of Sun et al [43] IL-6 prevented ConA-induced hepatitis via the suppression of NKT cells. This effect was partly due to the suppression of Fas ligand expression on NKT cells. Furthermore, adoptive transfer of hepatic mononuclear cells restored ConA-induced liver in jury in anti NK1.1 treated mice. However, adoptive transfer of hepatic mononuclear cells treated with IL-6 failed to restore such injury [43]. Taken together from our investigation, it is apparent that the presence and activity of NKT cells are needed for proper liver regeneration. Impairment of this process by ConA does not change this trend. Further studies are warranted to explore specifically the role of the multiple types of NKT cells. We also show that the impaired Cyclin B1 and p21 expression within the absence of NKT cells or when NKT cells are hampered is in conjugation with reduced liver regeneration and IL-6 levels.

## Conclusions

NKT cells are needed for liver regeneration. Impairment of Cyclin B1 and p21 expression in the absence of these cells is associated with reduced liver regeneration and serum IL-6.

## Abbreviations

ALT: Alanine aminotransferase
BrdU: Bromodeoxy uridine
ConA: Concanavalin A
IL-6: Intreleukin-6
iNKT: invariant NKT
MHC: Major histocompatibility complex
NKT: Natural killer T
PCNA: Proliferating cell nuclear antigen
PH: Partial hepatectomy
TNF: Tumor necrosis factor
